# Multiple morbidities in pregnancy: Time for research, innovation, and action

**DOI:** 10.1371/journal.pmed.1002665

**Published:** 2018-09-25

**Authors:** James G. Beeson, Caroline S. E. Homer, Christopher Morgan, Clara Menendez

**Affiliations:** 1 Burnet Institute, Melbourne, Victoria, Australia; 2 Central Clinical School and School of Public Health and Preventive Medicine, Monash University, Melbourne, Australia; 3 Department of Medicine and Melbourne School of Population and Global Health, University of Melbourne, Melbourne, Australia; 4 Centre for Midwifery, Child and Family Health, Faculty of Health, University of Technology Sydney, Sydney, Australia; 5 Barcelona Institute for Global Health (ISGlobal), Hospital Clinic of Barcelona, Universitat de Barcelona, Barcelona, Spain; 6 Centro de Investigação em Saúde de Manhiça, Maputo, Mozambique

## Abstract

In a Guest Editorial, James Beeson and colleagues discuss the contribution of nonobstetric morbidity to mortality during and around pregnancy and what needs to be done to address this global health challenge.

Progress indicators in maternal health in many low- and middle-income countries (LMICs) continue to fall below international standards despite Millennium Development Goal commitments and Sustainable Development Goal (SDG) aspirations [1]. While maternal mortality has fallen by 44% globally since 1990, many countries will struggle to meet the SDG target of fewer than 70 maternal deaths per 100,000 live births by 2030 [2]. Despite substantial efforts, globally over 300,000 women still die each year during pregnancy, childbirth, or the postpartum period, mostly from preventable causes. The burden of morbidity and mortality is inequitable, with vulnerable and marginalized populations at greatest risk. Although this burden disproportionately occurs in LMICs, it also affects increasing numbers of women in some high-income countries [3]. Improved counts of so-called indirect causes highlight the importance of nonobstetric morbidity during pregnancy, contributing around one-third of maternal deaths in LMICs [4]. Indirect causes include the effects of infections, noncommunicable diseases (NCDs), and mental health disorders. These highly prevalent conditions overlap and co-occur such that many women experience multimorbidity during and around pregnancy [5].

## Recognizing multimorbidity as an issue affecting pregnant women

Infectious diseases (such as HIV, malaria, tuberculosis, and sexually transmitted infections [STIs]) together with NCDs that are increasingly common in LMICs as a result of demographic, socioeconomic, and environmental changes (cardiovascular disease, diabetes, anemia, micronutrient deficiencies, hypertension, and mental health challenges) each account for substantial morbidity in pregnancy; however, the burden of their combination is less well recognized, and the impacts are not well understood. Some co-occurring morbidities have been well described, such as coinfection with HIV, malaria, or tuberculosis. Curable STIs (syphilis, gonorrhea, chlamydia, trichomonas, and others) likely infect about 40% of pregnant women in sub-Saharan Africa [6], with negative implications for the health of mother and baby. Infectious plus noninfectious multimorbidity is also important, such as malaria with anemia from nutritional deficiencies and tuberculosis with diabetes [1,5]. In LMIC settings, typically most pregnant women are anemic and have significant nutritional deficiencies [1,5], which may have impact on infections and NCDs [7]. Other multimorbidities are likely to have major impacts but are poorly understood. There is a major knowledge gap for many important co-occurring diseases and risk factors, especially the interaction of macro- and micronutrient deficiencies with infections and other conditions. In addition, poor maternal mental health has short- and long-term adverse effects on mothers and babies and has a likely impact on the whole family [8].

## Need for research and innovation

Addressing the burden of multimorbidity requires innovation and investment to drive the development of new tools, interventions, and strategies. We have listed the main priorities for research and innovation across levels of healthcare in Box 1. Greater research and innovation are urgently needed to identify common contributing factors and adversely synergistic interactions. Given the increasing burden of NCDs, there is a strong need to understand the interactions between different NCDs, as well as between infections and NCDs, among pregnant women. Clinical research is needed to test the potential of integrated interventions against common comorbidities (e.g., malaria and STIs, including HIV). Testing of interventions should be accompanied by improved surveillance and reporting of multimorbidities and a better understanding of the causes of maternal mortality, especially indirect deaths.

**Box 1. Priorities in research and innovation to address multimorbidities in pregnancy.**

1. Understand the major comorbidities, their interactions, cocontributing factors, and common pathways of diseases that co-occur in pregnancy, as well as interactions between drugs used to treat common conditions
2. Better understand and quantify long-term consequences for children and mothers of multimorbidity in pregnancy
3. Wider inclusion of pregnant women in clinical research and implementation studies
4. Develop new tools for diagnosis and screening for multimorbidities that are low cost, easy to use, and rapid and deployable in clinics and community settings within the available workforce
5. Develop and test new multimorbidity interventions informed by knowledge of interactions, common pathways, and root causes
6. Identify health service integration strategies that address multimorbidity in the antenatal, postnatal, and infancy periods
7. Develop models of care and a health workforce that promote health system–patient–community partnerships for care and interventions across the spectrum of reproductive, maternal, and child health
8. Promote greater investment by industry and biotech in technologies and products for maternal health (including diagnostics, therapeutics, vaccines, and mobile health [mHealth])

Determining pathogenic interactions underlying multimorbidity will be important to guide new interventions, and it is possible that, at least for some conditions, there may be common or interacting pathways that could be targeted (e.g., malaria in pregnancy together with undernutrition leading to low L-arginine levels can compound fetal growth restriction [9]). The use of systems biology approaches and application of platform technologies such as genomics, proteomics, and metabolomics may reveal pathogenesis mechanisms and better define processes to inform interventions and identify biomarkers for diagnostics or risk stratification. It is likely that there are common root causes and contributors for many conditions, such as low education, poverty, conflict and displacement, and fragile or nonfunctional health systems. Research to build knowledge on long-term consequences of multimorbidities in pregnancy for mothers and children is a priority to maximize potential health gains. New diagnostics and screening technologies for conditions that contribute to multimorbidity need to be brought within reach of LMICs: low cost, easy to use, rapid, and deployable in front-line clinics or community settings. Past examples include rapid diagnostic tests (RDTs) for specific infections, glycometers for diabetes, and urine test sticks, to name a few. Nucleic acid amplification technologies for highly sensitive testing of infections or risk factors are becoming simpler to use, more portable, and cheaper and may offer future solutions [10].

To achieve these advances also requires greater investment by industry and biotech in technologies and products for maternal health (including diagnostics, therapeutics, vaccines, and mHealth). Current levels of investment in research and development (R&D) in this field are very low, with very few new therapeutics, vaccines, or diagnostics for maternal health conditions being licensed in recent decades. Pregnant women are usually excluded from clinical trials of medical products, which has limited their access to the benefits of medical advances. Public–private partnerships and cofunding or incentive schemes could be used to enhance R&D investment, as has proven successful in several diseases. We argue that there should be a wider inclusion of pregnant women in clinical and implementation research; this will provide pregnancy-specific knowledge and help integrate maternal health into broader health solutions.

## Challenges for health services

Multimorbidity calls for new or enhanced models of care, as well as the application of new technologies and interventions. Innovative diagnostics and systems research will enable broader screening of pregnant women for diseases or risk factors, especially diseases that are frequently asymptomatic and have major impacts, such as curable STIs, poor nutrition, HIV, malaria, and others. Multimorbidity also demands more integrated service delivery, providing quality care that addresses the full spectrum of health needs of each woman; however, current evidence on how best to integrate pregnancy interventions into primary healthcare or public health programs, such as immunization programs, is insufficient to guide policy [11]. Also critical is integration through to postnatal care, which currently has unacceptably low coverage in LMICs and is typically provided through models that pay little attention to maternal multimorbidity [12]. Postnatal visits and even infant immunization visits represent key opportunities for further screening and follow-up of maternal conditions identified in pregnancy and childbirth.

Essential and just as urgent is the development and enabling of a sustainable health workforce that is trained for the challenges posed by multimorbidity. Significant shortages in the global health workforce, especially midwives and obstetricians, including access to continuing professional development, severely constrain the system’s capacity to provide quality care. Quality maternal and newborn care needs an emphasis on accessible and respectful care, prevention, and management of complications within a functional health system [13].

## Conclusion

The burden of multimorbidity in pregnancy, combined with global epidemiological changes in disease patterns and resource and health system constraints, poses challenges that can best be addressed by accelerating sustainable improvements in maternal health. Renewed and strengthened focus, research and innovation, and investment and partnerships to address this challenge are urgently needed.

## Abbreviations

LMIC: low- and middle-income country
mHealth: mobile health
NCD: noncommunicable disease
RDT: rapid diagnostic test
R&D: research and development
SDG: Sustainable Development Goal
STI: sexually transmitted infection
